# Extreme Precipitation and Beach Closures in the Great Lakes Region: Evaluating Risk among the Elderly

**DOI:** 10.3390/ijerph110202014

**Published:** 2014-02-14

**Authors:** Kathleen F. Bush, Cheryl L. Fossani, Shi Li, Bhramar Mukherjee, Carina J. Gronlund, Marie S. O’Neill

**Affiliations:** 1 Department of Environmental Health Sciences, School of Public Health, University of Michigan, 1415 Washington Heights, Ann Arbor, MI 48109, USA; E-Mails: gronlund@umich.edu (C.J.G.); marieo@umich.edu (M.S.O.); 2 Center for the Environment, Plymouth State University, 17 High St. Plymouth, NH 03264, USA; 3 Department of Epidemiology, School of Public Health, University of Michigan, 1415 Washington Heights, Ann Arbor, MI 48109, USA; E-Mail: cheryl.fossani@gmail.com; 4 Department of Biostatistics, School of Public Health, University of Michigan, 1415 Washington Heights, Ann Arbor, MI 48109, USA; E-Mails: shili@umich.edu (S.L.); bhramar@umich.edu (B.M.)

**Keywords:** aged, bathing beaches, climate change, Great Lakes region, gastrointestinal diseases, rain

## Abstract

As a result of climate change, extreme precipitation events are expected to increase in frequency and intensity. Runoff from these extreme events poses threats to water quality and human health. We investigated the impact of extreme precipitation and beach closings on the risk of gastrointestinal illness (GI)-related hospital admissions among individuals 65 and older in 12 Great Lakes cities from 2000 to 2006. Poisson regression models were fit in each city, controlling for temperature and long-term time trends. City-specific estimates were combined to form an overall regional risk estimate. Approximately 40,000 GI-related hospital admissions and over 100 beach closure days were recorded from May through September during the study period. Extreme precipitation (≥90th percentile) occurring the previous day (lag 1) is significantly associated with beach closures in 8 of the 12 cities (*p* < 0.05). However, no association was observed between beach closures and GI-related hospital admissions. These results support previous work linking extreme precipitation to compromised recreational water quality.

## 1. Introduction

The concentration of bacterial indicators in recreational water, such as *Escherichia coli* (*E. coli*), has been linked to cases of waterborne disease [1,2,3]. Health risks associated with exposure to contaminated recreational water include skin, eye, ear, and upper respiratory irritations and infections, as well as gastrointestinal illness (GI) [4]. Populations that may be at greater risk for contracting GI from contaminated recreational water include children, the elderly, and individuals with compromised immune systems [5]. 

The 1986 United States Environmental Protection Agency (EPA) recreational water quality criteria for freshwater beaches include a daily *E. coli* concentration of less than 235 colony forming units (CFUs) per 100 milliliters of water [2,3,4,5,6]. Bacteria concentrations exceeding these criteria trigger swimming advisories and/or beach closures to prevent exposure to waterborne pathogens. 

Recreational water can be contaminated from both point and nonpoint sources [7,8]. Additionally, recreational water quality is influenced by precipitation and other hydrometeorological parameters [9,10]. Precipitation is positively correlated with *E. coli* concentrations in recreational water [11,12,13]. High concentrations of fecal indicator-bacteria have been linked to GI-related health risks [14], specifically *E. coli* concentrations in freshwater [15].

Heavy precipitation and subsequent stormwater runoff can flush pathogens and other microorganisms directly into nearby surface water, resulting in increased concentrations of bacteria, and increased risk of waterborne disease [16,17,18]. Curriero *et al.* [16] observed that between 1948 and 1994, 51 percent of waterborne outbreaks occurring in the U.S. were preceded by precipitation above the 90th percentile. Additionally, Rose *et al.* [19] observed that between 1971 and 2004, 20 to 40 percent of outbreaks occurring in the U.S. were associated with precipitation above the 90th percentile. 

Previous studies have also reported a delayed onset of diarrheal disease following heavy rainfall events [16,20,21,22]. One explanation for the observed lag could be that the incubation period of waterborne pathogens ranges from one day, for pathogens such as *Shigella, Salmonella*, and *Rotavirus,* to two weeks for pathogens such as *Cryptosporidium* and *E. coli* [23,24]. In general, cases of GI peak within seven days of exposure to contaminated water [25,26].

Under predicted climatic changes, more extreme rain events are expected to occur, particularly in the Great Lakes region, which may increase the risk of poor recreational water quality [17]. Few epidemiological studies have looked at the effects of precipitation on beach closures and subsequent human health outcomes using time series analysis. This study investigates the association between beach closures and GI-related hospital admissions, comparing multiple smoothing approaches to control for long-term time trends.

While swimmers may be directly impacted by poor recreational water quality, elderly non-swimmers may be exposed to pathogens via drinking water as a result of increased turbidity following extreme events [22]. Our goal was to characterize the link between extreme precipitation and human health, by first evaluating the association between extreme precipitation and beach closures, and subsequently evaluating the risk of GI-related hospital admissions among the elderly as a function of both extreme precipitation and the occurrence of beach closures. Because recreational water quality data were only available during summer months, we introduce an innovative method to control for long-term time trends in the discontinuous data and evaluate the potential bias of using a discontinuous time-series.

## 2. Experimental Section

### 2.1. Study Location

The U.S. Great Lakes region provides approximately 40 million people with water used for drinking, fishing, recreation, and industry [17,27]. The region encompasses over 1,000 beaches and 5,500 miles of shoreline [28]; currently the region experiences the highest percentage of beach closures as a result of poor water quality compared to other regions in the U.S. [29]. This study includes both inland beaches and beaches along the Great Lakes in the Great Lakes region.

**Figure 1:**
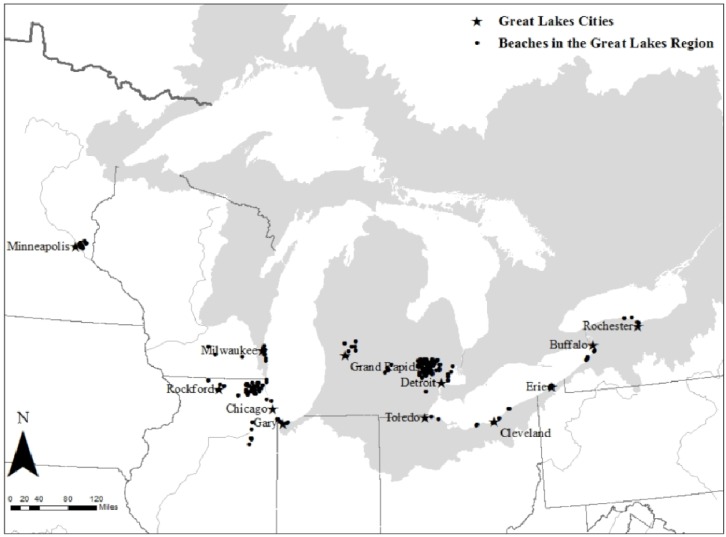
Location of beaches in the Great Lakes region included in this analysis, cities correspond to the surrounding county or counties for which data was available.

This study focused on 12 cities within the Great Lakes region for which sufficient beach closure data were available (Table 1; Figure 1). To examine city-specific associations, county-level beach closure data were matched to the corresponding Metropolitan Statistical Area, thus forming the cities used in this analysis. The majority of cities correspond to only one county, however, larger cities (*i.e.*, Chicago, IL; Cleveland, OH; and Detroit, MI) correspond to several surrounding counties. These metropolitan areas represent the core urban area and surrounding suburbs that have a high degree of social and economic integration.

**Table 1:** Cities in the Great Lakes region included in this analysis, defined as the county or counties surrounding the Metropolitan Statistical Area.

City	State	County
Buffalo	NY	Erie
Chicago	IL	Cook
		Lake
		McHenry
		Will
Cleveland	OH	Cuyahoga
		Lake
		Lorain
Detroit	MI	Macomb
		Oakland
		Wayne
Erie	PA	Erie
Gary	IN	Lake
Grand Rapids	MI	Kent
Milwaukee	WI	Milwaukee
Minneapolis	MN	Ramsey
Rochester	NY	Monroe
Rockford	IL	Winnebago
Toledo	OH	Lucas

### 2.2. Data Sources and Variables for Analysis

#### 2.2.1. Hospital Admissions

Hospital admission (HA) records for individuals 65 years and older and enrolled in Medicare were obtained from the Centers for Medicare and Medicaid Services for the 12 cities from 2000 to 2006. Approximately 98 percent of all people in this age range are enrolled in Medicare [30]. Hospital admission records included date of admission, cause of admission (International Classification of Disease, 9th Revision, ICD-9), and individual-level characteristics, including patient age, sex, race, and zipcode. University of Michigan Institutional Review Board approval was obtained for this analysis.

Based on previous research, cause of admission was defined as GI-related if the primary, secondary, or tertiary ICD-9 code was classified as: (i) a pathogen specific intestinal infectious disease (ICD 001-007; 120-129), (ii) other and ill-defined intestinal infectious disease (008–009), or (iii) diarrheal disease-related symptoms (276, 558.9, 787) [22,31]. Data were collapsed into daily counts of GI for each of the 12 cities. 

#### 2.2.2. Beach Closures

Daily recreational water quality data were obtained from the county-level organizations responsible for water quality monitoring in each of the 12 cities. In some cases these data were publicly available, but in other instances the data were accessed via direct communication with Recreational Water Quality and Beach Program Managers. Data included daily concentration of *E. coli* or fecal coliform bacteria in water samples during the summer swimming period (1 May–30 September) over the study period 2000–2006. A beach was defined as closed if the concentration of *E. coli* was greater than or equal to the full body contact (single sample) standard of 235 CFUs per 100 mL of water or if the concentration of fecal coliforms was greater than or equal to the geometric mean standard of 200 CFUs per 100 mL of water [6]. Otherwise, a beach was defined as open. Although the EPA water quality criteria were updated in 2012 to increase protection of primary contact recreation, in the current study beach closures were defined based on previously defined criteria. 

When more than one measure of bacterial concentration was reported for one beach on a single day, a daily average concentration was used. Because the number of beaches monitored on a daily basis varied by city and year, a binary variable was created to describe whether a recreational water quality advisory was administered, which allowed for standardization across cities. This variable took the value of 1 if any beach within the city was closed on a particular day and 0 if all beaches within the city were open. In Chicago and Rockford, IL water quality data were only available as a list of dates when beach closures occurred. Days when one or more beaches were closed within the city were coded as 1. All other weekdays, beaches were assumed to be open and were coded as 0. Data were not imputed for weekend days and were left as missing when no date was listed. Although this analysis modeled beach closures as a binary indicator variable, the underlying decision to close a beach was based on the actual bacterial concentration measured in the water.

#### 2.2.3. Meteorological Conditions

Hourly meteorological data including precipitation, temperature, dew point, and relative humidity were downloaded from the first order weather station of the National Weather Service (NWS) Cooperative Observer Program [32] for each city. Apparent temperature (AT) was used as a measure of the combined effects of temperature and humidity: AT = −2.653 + (0.994 × T_a_) + (0.0153 × T_d_^2^), where T_a_ is equal to air temperature (°C) and T_d_ is equal to dew point temperature (°C) [33,34]. Daily summaries were created from the hourly measurements for apparent temperature and total precipitation. 

Precipitation was categorized based on the city-specific summer time rainfall distribution. Categories were defined as: (1) Precipitation equal to 0 (reference category); (2) greater than 0, but less than 0.01 inches (0.25 mm); (3) greater than or equal to 0.01 inches (0.25 mm), but less than the 90th percentile and; (4) greater than or equal to the 90th percentile. Thus, the effects of no, trace, moderate, and extreme precipitation were evaluated. The 90th percentile was chosen as the cutoff for extreme precipitation based on previous research and the observable increase in risk of water contamination during extreme precipitation events [9,13,35]. Table 2 lists the data sources for hospital admissions, recreational water quality, and meteorological data used in this analysis.

**Table 2:** Data sources corresponding to hospital admission, meteorological, and recreational water quality data.

Data Type	Data Source
*Hospital Admission Data*	Centers for Medicare and Medicaid Services
*Meteorological Data*	National Weather Service Cooperative Observer Program
*Recreational Water Quality Data* (county, state)	
Cook; Lake; McHenry; Will; and Winnebago, IL	Illinois Department of Public Health: Environmental Health
Lake, IN	Indiana Department of Environmental Management
Kent; Macomb; Oakland; and Wayne, MI	Michigan Department of Natural Resources and the Environment
Ramsey, MN	Ramsey County Public Works
Erie; and Monroe, NY	New York State Health Department
Cuyahoga; Lake; Lorain; and Lucas, OH	Ohio Department of Health
Erie, PA	Erie County Department of Health
Milwaukee; and Waukesha, WI	Wisconsin Department of Natural Resources

### 2.3. Statistical Analysis

The primary goal of this study was to estimate the association between extreme precipitation and beach closures, and subsequent risk of GI-related hospital admissions, while controlling for meteorological conditions. In cases, like this, where only a certain season is of interest (e.g., summer), it is common to use a discontinuous time-series to splice together the seasons of interest over the study period. This method forces the estimate at the end of the season of interest in one year to match the estimate at the beginning of the season in the following year, without regard to effects of the “off season” on the estimate. Therefore, a secondary goal of this study was to evaluate potential bias associated with using summer-only data in time-series analysis and introduce innovative methods to reduce such bias. In our study, Poisson regression models were fit under three scenarios to control for long-term time trends in the data. First, models were run without using a spline term; second, with a spline term estimated by the discontinuous summer-only time-series; and thirdly, using a two-stage Poisson regression approach. In the two-stage approach, the spline term was initially estimated using the entire hospital admission time-series. The estimated spline fragments corresponding to the seven summers were then added to the Poisson regression model as an offset.

City-specific statistics were summarized using scatterplots and histograms. In order to compare our data to previous research, which found precipitation during the previous 1–3 days to be a strong predictor of recreational water quality, a city-specific logistic regression was used to estimate the association between precipitation (PRCP) and beach closures (BC) over a 3-day lag period (Model 1) [9,36,37]:

Model 1: [logit[Pr(BC=1)] = β_0_ + β_ 1_PRCP_lag-x_]

where BC is a binary variable representing the occurrence of a beach closure and precipitation is a categorical variable based on the 90th percentile, x = 1, 2, and 3. Next, the crude association between beach closures and daily GI-related hospital admissions was evaluated using city-specific Poisson regression models. The over-dispersion parameter was considered [38] and tested using Dean’s test [39].

#### 2.3.1. Exploring Lags

Because observed health effects may occur several days after exposure due to delayed onset of clinical symptoms and environmental transport, a 7-day lag period was chosen for this analysis to be consistent with the incubation period of most bacterial and viral waterborne pathogens [40]. Seven separate models were run using precipitation at single-day lags, 1 to 7 days prior to the hospitalization date, as the independent variable. 

#### 2.3.2. Exploring Confounding

As apparent temperature can influence pathogen replication, persistence, and transmission [26,41,42,43] as well as the health of elderly populations [44], it was included as a potential confounder. It was matched to the lagged day of beach closure. Because hospital admissions are known to vary by day of week, an indicator variable for day of week was also included in the model (Model 2):

Model 2: log[E(HA_t_)] = β_0_ + β_1_BC_t-q_ + β_2_PRCP_t-q_ + β_3_AT_t-q _+ β_4_DOW_t_

where HA is daily GI-related hospital admissions, BC is a binary variable representing the occurrence of a beach closure, precipitation is categorized based on the 90th percentile, q = 1, 2, … 7 represents single-day lags 1–7 days prior to the day of hospital admission, AT represents apparent temperature, and DOW represents the day of week.

#### 2.3.3. Exploring the Effect of Long-Term Time Trends

To control for long-term time trends in hospital admissions, a nonlinear smoothing term for time was included in the Poisson regression model (*i.e.*, penalized spline). Smoothing parameters were estimated to minimize the generalized cross validation score [45,46]. This model took the 7-year summer-only time-series and spliced the summer periods together, creating a discontinuous time-series (Model 3):

Model 3: log[E(HA_t_)] = β_0_ + β_1_BC_t-q_ + β_2_PRCP_t-q_ + β_3_AT_t-q _+ β_4_DOW_t_ + s(t)

where s(time) represents a penalized spline on time. 

The final stage of analysis was a two-stage Poisson regression model, in which the entire 7-year time-series of GI-related hospital admissions was fit using a penalized spline term (Model 4, Stage 1). The estimated spline fragments corresponding to the seven summers were then added to the full Poisson regression model as an offset. This model also explored lags and potential confounders (Model 4, Stage 2):

Model 4, Stage 1: log[E(HA_t_)] = s(t)
Model 4, Stage 2: log[E(HA_t_)] = β_0_ + β_1_BC_t-q _+ β_2_PRCP_t-q_ + β_3_AT_t-q_ + β_4_DOW_t_ + offset_t_

#### 2.3.4. Calculating A Regional Estimate

City-specific estimates were collapsed into an overall summary estimate for the region using a fixed-effect model. Results were pooled using the inverse-variance weighting estimator. If the null hypothesis was rejected (*p* ≤ 0.05), a random-effects model, accounting for both within- and between-city variation, was applied [47,48]. All analyses were run using SAS Version 9.2 (SAS Institute, Cary, NC, USA) and R 12.0 (R Foundation for Statistical Computing, Vienna, Austria). 

## 3. Results and Discussion

Over the 7-year study period, approximately 40,000 GI-related hospital admissions were recorded among individuals over the age of 65 across 12 cities in the Great Lakes region (Table 3). The average number of daily GI-related hospital admissions ranged from 0.42 in Erie, PA to 14.47 in Chicago, IL, with an overall daily average of 2.66. From 2000 to 2006, over 100 beach closure days were recorded during the swimming season, defined as May 1st to September 30th. On average, beaches were closed 10% of the time. However, in Chicago, IL; Cleveland, OH; and Milwaukee, WI, beaches were closed over 20% of the time. Daily precipitation during the swimming season in the Great Lakes region ranged from 0 to 4.45 inches (113 mm), with an overall mean daily total of 0.12 inches (3.05 mm). For all 12 cities, precipitation had a skewed distribution, with zero precipitation recorded on nearly 65% of days during the swimming season. Mean daily apparent temperature, for the region, was 19 °C (67 °F). Precipitation and apparent temperature followed consistent seasonal trends throughout the study period across all cities. 

Extreme precipitation above the 90th percentile, occurring on the previous day (lag 1), was a significant predictor (*p* < 0.05) of beach closures in 8 of the 12 cities (Buffalo, NY; Cleveland, OH; Detroit, MI; Erie, PA; Gary, IN; Milwaukee, WI; Rochester, NY; and Toledo, OH) (Table 4). However, no consistent trends were observed for the risk of GI-related hospital admissions following a beach closure (Table 5). In Erie, PA; Minneapolis, MN; Rochester, NY; and Toledo, OH beach closures were positively associated with GI-related hospital admissions among the elderly in at least one of the 7 different lag models. In Buffalo, NY; Chicago, IL; Cleveland, OH; and Detroit, MI, however, the association between beach closures and GI-related hospital admissions indicated a statistically significant inverse relationship in at least one of the seven different lag models. In the four remaining cities Gary, IN; Grand Rapids, MI; Milwaukee, WI; and Rockford, IL no significant associations were found.

In the instances where beach closures were positively associated with GI-related hospital admissions, lags 1, 2, 3, and 7 were significant. Risk ratios ranged from 1.30 (95% confidence interval (CI): 1.00, 1.68) in Rochester at lag 3 to 1.76 (95% CI: 1.13, 2.75) in Minneapolis at lag 1. As a sensitivity analysis, models were re-run with an indicator of cumulative exposure to extreme precipitation (7-day moving average); results were consistent. When the results were pooled across the 12 cities, the overall effect estimate was not significant (Table 5). The main effect of extreme precipitation on hospital admissions was only significant in two cities: in Chicago at lag 1 with a RR of 1.12 (1.00, 1.24) and in Detroit at lag 6 with a RR of 1.29 (1.06, 1.58) (Appendix Table A1). The lack of statistical significance may be due to low power related to too few extreme events or too few GI-related hospital admissions.

**Table 3:** Summary statistics for 12 Great Lakes cities during the swimming season (1 May–30 September) from 2000 to 2006.

City	Population Over 65^a^ (% of Population)	Mean Daily GI-Related Admissions (per 100,000)	Mean Daily Beach Closures (Total)	Median daily Total Precipitation (mm) (90th Percentile)	Mean daily Apparent Temperature °C (°F)
Buffalo, NY	151,258 (16)	1.48 (0.98)	0.93 (292)	0.00 (9.40)	18.99 (66.19)
Chicago, IL	747,777 (11)	14.47 (1.94)	0.61 (506)	0.00 (9.63)	20.39 (68.71)
Cleveland, OH	284,788 (15)	4.89 (1.72)	1.47 (535)	0.00 (9.63)	20.22 (68.39)
Detroit, MI	491,592 (12)	7.35 (1.50)	0.71 (342)	0.00 (9.40)	20.44 (68.80)
Erie, PA	40,256 (14)	0.42 (1.04)	0.40 (103)	0.00 (10.67)	19.38 (66.89)
Gary, IN	63,234 (13)	0.95 (1.50)	0.90 (293)	0.00 (10.67)	20.27 (68.49)
Grand Rapids, MI	59,625 (10)	0.69 (1.16)	0.43 (15)	0.00 (11.43)	19.14 (66.46)
Milwaukee, WI	121,685 (13)	2.38 (1.96)	0.90 (376)	0.00 (9.40)	19.06 (66.31)
Minneapolis, MN	59,502 (12)	1.95 (3.28)	0.23 (17)	0.00 (10.67)	19.33 (67.79)
Rochester, NY	95,779 (13)	0.80 (0.84)	0.40 (145)	0.00 (9.65)	19.29 (66.22)
Rockford, IL	35,450 (13)	0.51 (1.44)	0.10 (75)	0.00 (9.65)	20.14 (68.26)
Toledo, OH	59,441 (13)	0.57 (0.96)	0.44 (115)	0.00 (9.65)	20.44 (68.8)

Note: ^a^ Population estimate based on the 2000 U.S. Census [49].

**Table 4:** City-specific odds ratios (OR) with p-values evaluating the association between daily categorical precipitation ^a^ at lag 1 (1-day previous) and beach closures in 12 Great Lakes cities from 2000 to 2006.

Precipitation Category	City-specific OR	City-specific OR	City-specific OR	City-specific OR
	(*p*-value)	(*p*-value)	(*p*-value)	(*p*-value)
	*Buffalo, NY*	*Chicago, IL*	*Cleveland, OH*	*Detroit, MI*
0 < prcp < 0.01	2.42 (0.14)	1.69 (0.23)	1.77 (0.30)	1.28 (0.68)
0.01 ≤ prcp < 90th percentile	2.94 (<0.001)	1.34 (0.14)	1.65 (0.07)	1.42 (0.13)
prcp ≥ 90th percentile	16.93 (<0.001)	1.20 (0.41)	7.39 (0.00)	4.02 (<0.001)
	*Erie, PA*	*Gary, IN*	*Grand Rapids, MI*	*Milwaukee, WI*
0 < prcp < 0.01	0.00 (0.98)	1.48 (0.70)	-	0.93 (0.89)
0.01 ≤ prcp < 90th percentile	2.31 (0.09)	1.53 (0.15)	1.71 (0.54)	1.41 (0.22)
prcp ≥ 90th percentile	10.21 (<0.001)	2.01 (0.05)	0.57 (0.64)	2.01 (0.04)
	*Minneapolis, MN*	*Rochester, NY*	*Rockford, IL*	*Toledo, OH*
0 < prcp < 0.01	2.00 (0.59)	2.67 (0.03)	0.00 (0.09)	2.02 (0.29)
0.01 ≤ prcp < 90th percentile	1.33 (0.75)	1.91 (0.03)	0.51 (0.17)	1.24 (0.55)
prcp ≥ 90th percentile	1.60 (0.50)	5.67 (<0.001)	0.66 (0.40)	9.07 (<0.001)

Not: ^a^ Reference category is where precipitation is equal to 0.

**Table 5:** City-specific risk ratios ^a^ (95% confidence intervals) corresponding to the risk of GI-related hospital admissions among the elderly following beach closures over a 1-week lag using a two-stage spline structure in 12 Great Lakes cities 2000–2006.

	*Buffalo, NY*	*Chicago, IL*	*Cleveland, OH*	*Detroit, MI*	*Erie, PA*
lag 1	0.96 (0.79, 1.16)	0.96 (0.91, 1.00)	0.99 (0.90, 1.09)	1.01 (0.94, 1.08)	1.49 (0.90, 2.46)
lag 2	0.97 (0.79, 1.19)	1.02 (0.97, 1.07)	1.05 (0.95, 1.17)	1.00 (0.93, 1.08)	1.67 (1.02, 2.76)
lag 3	1.04 (0.85, 1.28)	1.00 (0.95, 1.05)	0.88 (0.80, 0.98)	0.97 (0.90, 1.05)	1.15 (0.69, 1.93)
lag 4	0.98 (0.81, 1.20)	1.01 (0.96, 1.06)	0.96 (0.86, 1.06)	0.99 (0.92, 1.07)	1.23 (0.70, 2.18)
lag 5	0.78 (0.63, 0.96)	1.02 (0.97, 1.07)	1.02 (0.92, 1.14)	0.92 (0.86, 0.99)	0.49 (0.22, 1.06)
lag 6	0.92 (0.75, 1.12)	1.02 (0.98, 1.08)	1.03 (0.93, 1.15)	0.95 (0.88, 1.02)	1.54 (0.89, 2.65)
lag 7	0.92 (0.75, 1.12)	1.00 (0.96, 1.05)	0.96 (0.87, 1.06)	0.97 (0.90, 1.04)	0.94 (0.52, 1.68)
	*Gary, IN*	*Grand Rapids, MI*	*Milwaukee, WI*	*Minneapolis, MN*	*Rochester, NY*
lag 1	0.90 (0.71, 1.15)	0.70 (0.22, 2.13)	1.05 (0.89, 1.24)	1.76 (1.13, 2.75)	0.84 (0.64, 1.10)
lag 2	1.08 (0.85, 1.38)	1.74 (0.74, 4.09)	1.02 (0.87, 1.20)	1.13 (0.72, 1.75)	0.86 (0.65, 1.12)
lag 3	1.01 (0.80, 1.28)	1.13 (0.51, 2.51)	0.99 (0.84, 1.17)	1.08 (0.68, 1.69)	1.30 (1.00, 1.68)
lag 4	1.03 (0.81, 1.31)	1.26 (0.50, 3.17)	1.03 (0.88, 1.21)	0.70 (0.40, 1.22)	0.96 (0.73, 1.26)
lag 5	0.99 (0.78, 1.25)	0.66 (0.17, 2.57)	1.08 (0.92, 1.27)	1.14 (0.69, 1.86)	0.97 (0.74, 1.28)
lag 6	1.11 (0.87, 1.41)	1.49 (0.49, 4.50)	0.99 (0.84, 1.16)	1.10 (0.73, 1.67)	1.03 (0.79, 1.35)
lag 7	0.87 (0.69, 1.11)	2.41 (0.75, 7.77)	1.07 (0.91, 1.26)	0.75 (0.51, 1.10)	1.19 (0.92, 1.53)
	*Rockford, IL*	*Toledo, OH*	*Pooled-Estimate*		
lag 1	1.11 (0.67, 1.82)	0.97 (0.68, 1.38)	0.98 (0.95, 1.01)		
lag 2	0.78 (0.42, 1.43)	0.70 (0.47, 1.02)	1.01 (0.98, 1.05)		
lag 3	0.83 (0.46, 1.50)	1.13 (0.77, 1.65)	0.98 (0.95, 1.02)		
lag 4	1.04 (0.62, 1.74)	0.64 (0.43, 0.97)	1.00 (0.96, 1.03)		
lag 5	1.35 (0.85, 2.13)	1.03 (0.71, 1.48)	0.99 (0.95, 1.02)		
lag 6	0.77 (0.42, 1.43)	1.01 (0.71, 1.45)	1.01 (0.97, 1.04)		
lag 7	1.30 (0.81, 2.10)	1.67 (1.22, 2.30)	0.99 (0.96, 1.03)		

Note: ^a^ Two-stage Poisson regression adjusted for meteorological conditions, day of week, and long-term time trends.

**Figure 2:**
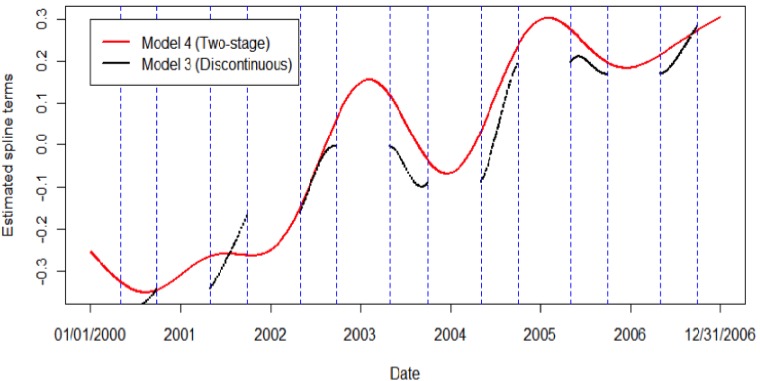
The discontinous, summer-only spline compared to the spline estimated using the entire 7-year time-series in the two-stage spline model, using Detroit, MI as an example.

Comparing the different spline structures, no significant differences were observed. In cities where a significant association was observed in at least one of the seven different lag models, that association was consistent across spline structures (Appendix Table A2). In cities where no association was observed for any lag, that also remained consistent across spline structures. While the different spline structures used to control long-term time trends did not alter the significance or magnitude of the associations reported, there was an observable difference between the two different spline estimates (Figure 2). Using Detroit as an example, the spline estimated from the discontinuous time-series was unique compared to the spline estimated from the entire time-series in the two-stage analysis. In all instances, the spline estimated from the entire time-series was numerically different from that estimated from the discontinuous time-series. 

In general, extreme precipitation, above the 90th percentile, at lag 1, was a significant predictor of beach closures in 8 of the 12 cities. However, no consistent association between beach closures and GI-related hospital admissions among the elderly was observed. In this study, novel methodology to control for long-term time trends using season-specific data was proposed and results using three different spline structures were compared. While no significant differences in the effect estimates were observed in this analysis, the two-stage Poisson model, which utilizes the full time-series to control for long-term time trends in the outcome variable, is recommended for future work focused on season-specific analyses. 

The two-stage spline structure presented in Model 4 can be applied to a variety of studies where only one season is of interest. By comparing results from the two-stage spline model to results from a model with no spline, as well as a model with a spline estimated from the discontinuous, summer-only time-series, we addressed an important methodological question regarding the most appropriate way to conduct time-series analysis when exposure data is only available for a portion of the year. Results, in this case, did not differ markedly across the three different modeling approaches. One explanation may be that GI-related hospital admissions did not display significant variability between summer, the season of interest, and the rest of the year. Differences in effect estimates are more likely to be observed between the discontinuous time-series model and a two-stage time-series model when the health outcome varies across seasons. If hospital admissions had shown more variability across seasons, the two-stage spline structure would have minimized confounding by long-term time trends and reduced potential bias.

Although the results presented here do not reveal a consistent or significant association between beach closures and GI-related hospital admissions, previous research states that poor recreational water quality has the potential to adversely impact human health. Previous research confirms that precipitation is linked to water quality indicators such as *E. coli* concentrations and turbidity [9,36]. *E. coli* concentrations in recreational waters are estimated to peak approximately 24 to 72 hours following precipitation events in the Great Lakes region [11,13]. Previous research has also reported a delayed onset of diarrheal disease following extreme precipitation and related increases in water quality indicators [16,19,21,22,50,51].

While Sampson *et al.* [52] found no association between rainfall and bacteria at any of their 15 sites along the Wisconsin shores of Lake Superior (water samples were taken following any rain event of at least 0.25 inches (6.35 mm)), our study evaluated extreme precipitation events, above the 90th percentile (0.40 inches (10.16 mm)). These extreme events were found to be a significant predictor of recreational water quality in a majority of cities. Results from a specific location should not necessarily be used to make decisions regarding beach closures at other locations [52]. For example, Haack *et al.* [36] concluded that rainfall 48 to 72 hours prior was significantly associated with *E. coli* concentrations at three Southern beaches in Grand Traverse Bay in Lake Michigan, but only 24 h prior at Western and Eastern beach locations.

Results from our analysis suggest that precipitation should be modeled in a way that accommodates the skewed distribution and the nonlinear associations often observed between precipitation and daily hospital admissions. Modeling precipitation as a categorical variable, as we did, is a suitable approach. 

One of the primary limitations of this analysis is related to data specificity; GI-related hospital admissions are dramatically underreported and the etiology is rarely identified [53,54]. The symptoms associated with exposure to contaminated recreational water are relatively broad-spectrum symptoms. Therefore, it is challenging to observe direct associations between exposure and outcome. Additionally, the period of interest is quite limited: on average only 35% of summer days had measurable amounts of precipitation. Further, recreational water quality monitoring was not consistent over the study period. Use of a binomial indicator for beach closings may have biased results towards the null. This binary coding also fails to differentiate between when one beach was closed and ten beaches were closed for a particular city, such misclassification may make it more difficult to identify a significant association. The absence of an association between extreme precipitation and GI-related hospitalization could also be due to infrequent use of and limited exposure to surface waters for recreation by Medicare beneficiaries.

Lastly, it is important to note that precipitation can be much localized; use of single city monitoring stations did not allow for spatially explicit analysis. However, a major strength of this analysis was its use of publicly available data across a wide geographic area to explore the impact of extreme precipitation on beach closures and subsequent risk of waterborne disease, which has implications for recreational water management at the local level.

This study was conducted to evaluate whether beach closure and Medicare data, both easily accessible, could be used as a proxy for evaluating risk of GI. The development of such a universal model would help beach managers and public health professionals assess risk across a wide geographic area and prioritize resources accordingly. Because the association between recreational water quality and hospital admissions was only being investigated in select cities in the Great Lakes region, conclusions may not be applicable to marine or estuarine recreational waters or other regions of the country where socio-demographic, meteorological, and hydrodynamic conditions may vary. 

Future work in this area should promote the use of a consistent definition of extreme precipitation so that decision-makers can have a shared understanding of the risks associated with heavy precipitation events. Our results linking extreme precipitation to beach closures provide additional support for precipitation-based public health warning systems [55].

## 4. Conclusions

In a majority of the 12 Great Lakes cities, extreme precipitation (≥90th percentile) was significantly associated with beach closures. However, no consistent trend was observed between beach closures and GI-related hospital admissions among the elderly. Nonetheless, the risk of waterborne disease outbreaks must be considered in the context of a changing climate. In order to predict future health outcomes, it is critical to understand how current meteorological factors drive seasonal patterns of water quality and disease [24]. According to the IPCC Special Report on managing the risks of extreme events and disasters, a changing climate is linked to changes in frequency, intensity, duration, and timing of extreme events [56]. These changes in precipitation will directly and indirectly impact runoff, which has the potential to impact pathogen transport. In particular, higher winter precipitation has the potential to increase the occurrence of flooding and combined sewer overflows, while increasing summer temperatures are likely to increase the hydrophobicity of soils, reducing infiltration rates [57]. Further investigation exploring the impacts of these climatic changes on pathogen concentrations in recreational waters, and the risk of waterborne disease outbreaks across seasons is warranted. Future work linking extreme precipitation to beach closures and GI-related hospital admissions should incorporate the role of combined sewer overflows (CSOs), proximity to river outlets, and land cover, which are likely to influence the abundance and transport of pathogens in the environment. Specifically, future work should investigate whether beach closures due to microbial contamination are more likely at beaches in close proximity to CSOs, downstream of heavily urbanized areas, or nearby agricultural land. Finally, enhanced monitoring and surveillance of recreational water quality and cases of waterborne disease will improve our ability to protect human health in the face of a changing climate.

## Appendix

**Table A1:** City-specific risk ratios (95% confidence intervals) corresponding to the risk of GI-related hospital admissions among the elderly following extreme precipitation over a 1-week lag using a two-stage spline structure in 12 Great Lakes cities 2000–2006.

	*Buffalo, NY*	*Chicago, IL*	*Cleveland, OH*	*Detroit, MI*	*Erie, PA*
lag 1	0.69 (0.38, 1.25)	1.12 (1.00, 1.24)	1.02 (0.83, 1.25)	0.98 (0.78, 1.20)	1.33 (0.38, 4.60)
lag 2	1.26 (0.80, 1.99)	0.97 (0.86, 1.09)	1.03 (0.84, 1.27)	0.92 (0.73, 1.15)	0.34 (0.05, 2.49)
lag 3	1.11 (0.67, 1.83)	1.02 (0.90, 1.15)	1.13 (0.93, 1.38)	1.04 (0.83, 1.30)	2.33 (0.94, 5.77)
lag 4	0.61 (0.33, 1.13)	0.99 (0.88, 1.12)	0.96 (0.77, 1.21)	1.05 (0.83, 1.31)	1.04 (0.30, 3.61)
lag 5	1.04 (0.62, 1.72)	0.99 (0.88, 1.11)	0.93 (0.74, 1.17)	0.78 (0.61, 1.00)	0.96 (0.22, 4.14)
lag 6	0.99 (0.59, 1.69)	1.05 (0.94, 1.18)	1.01 (0.82, 1.26)	1.29 (1.06, 1.58)	1.02 (0.23, 4.46)
lag 7	1.10 (0.68, 1.79)	1.10 (0.98, 1.23)	1.11 (0.91, 1.36)	1.02 (0.81, 1.27)	0.75 (0.11, 5.37)
	*Gary, IN*	*Grand Rapids, MI*	*Milwaukee, WI*	*Minneapolis, MN*	*Rochester, NY*
lag 1	1.14 (0.42, 3.11)	0.51 (0.13, 1.87)	0.86 (0.55, 1.35)	0.83 (0.27, 2.49)	1.25 (0.77, 2.02)
lag 2	1.52 (0.64, 3.59)	1.31 (0.53, 3.25)	0.91 (0.59, 1.39)	1.02 (0.41, 2.52)	0.80 (0.45, 1.41)
lag 3	0.42 (0.09, 2.00)	1.23 (0.55, 2.75)	0.93 (0.61, 1.43)	1.28 (0.60, 2.77)	1.18 (0.72, 1.94)
lag 4	1.30 (0.55, 3.07)	0.47 (0.14, 1.57)	1.19 (0.83, 1.73)	0.71 (0.23, 2.16)	1.09 (0.65, 1.84)
lag 5	1.45 (0.62, 3.39)	1.33 (0.44, 4.04)	0.66 (0.41, 1.06)	1.57 (0.67, 3.66)	0.86 (0.48, 1.56)
lag 6	0.91 (0.34, 2.49)	0.66 (0.18, 2.40)	1.02 (0.69, 1.52)	1.05 (0.45, 2.44)	1.21 (0.75, 1.95)
lag 7	0.90 (0.30, 2.72)	0.87 (0.23, 3.26)	1.08 (0.74, 1.58)	0.51 (0.20, 1.29)	1.00 (0.59, 1.72)
	*Rockford, IL*	*Toledo, OH*			
lag 1	1.50 (0.90, 2.53)	0.71 (0.30, 1.69)			
lag 2	0.79 (0.38, 1.61)	0.86 (0.39, 1.88)			
lag 3	1.20 (0.64, 2.24)	0.68 (0.27, 1.70)			
lag 4	1.02 (0.53, 1.96)	0.73 (0.30, 1.82)			
lag 5	0.66 (0.31, 1.43)	0.98 (0.46, 2.11)			
lag 6	0.97 (0.47, 2.00)	1.73 (0.95, 3.15)			
lag 7	1.44 (0.79, 2.63)	1.69 (0.94, 3.02)			

**Table A2:** City-specific risk ratios (95% confidence intervals) corresponding to the risk of GI-related hospital admissions among the elderly following beach closures over a 1-week lag using discontinuous time-series in 12 Great Lakes cities 2000–2006.

	*Buffalo, NY*	*Chicago, IL*	*Cleveland, OH*	*Detroit, MI*	*Erie, PA*
lag 1	0.94 (0.78, 1.14)	0.96 (0.91, 1.01)	1.02 (0.92, 1.12)	1.01 (0.94, 1.08)	1.35 (0.81, 2.25)
lag 2	0.96 (0.79, 1.18)	1.02 (0.97, 1.08)	1.08 (0.97, 1.19)	1.00 (0.93, 1.08)	1.49 (0.90, 2.49)
lag 3	1.04 (0.84, 1.27)	0.99 (0.94, 1.05)	0.89 (0.81, 0.99)	0.97 (0.90, 1.05)	1.09 (0.65, 1.84)
lag 4	0.96 (0.78, 1.17)	1.01 (0.96, 1.07)	0.97 (0.87, 1.08)	1.00 (0.93, 1.07)	1.24 (0.69, 2.20)
lag 5	0.76 (0.62, 0.93)	1.02 (0.97, 1.08)	1.04 (0.93, 1.16)	0.92 (0.86, 0.99)	0.48 (0.22, 1.05)
lag 6	0.89 (0.73, 1.09)	1.03 (0.98, 1.08)	1.06 (0.95, 1.17)	0.95 (0.88, 1.02)	1.58 (0.91, 2.74)
lag 7	0.91 (0.74, 1.11)	1.01 (0.97, 1.06)	0.97 (0.87, 1.08)	0.97 (0.90, 1.04)	0.87 (0.48, 1.58)
	*Gary, IN*	*Grand Rapids, MI*	*Milwaukee, WI*	*Minneapolis, MN*	*Rochester, NY*
lag 1	0.95 (0.74, 1.22)	0.62 (0.20, 1.93)	1.02 (0.86, 1.20)	1.84 (1.16, 2.91)	0.82 (0.63, 1.08)
lag 2	1.16 (0.90, 1.49)	1.89 (0.76, 4.68)	1.01 (0.86, 1.19)	1.09 (0.69, 1.71)	0.84 (0.64, 1.11)
lag 3	1.09 (0.85, 1.39)	1.11 (0.48, 2.60)	0.99 (0.84, 1.17)	0.96 (0.60, 1.55)	1.28 (0.99, 1.65)
lag 4	1.13 (0.88, 1.46)	1.37 (0.53, 3.58)	1.05 (0.90, 1.23)	0.61 (0.35, 1.05)	0.94 (0.72, 1.24)
lag 5	1.06 (0.82, 1.38)	1.48 (0.26, 8.57)	1.08 (0.92, 1.27)	1.10 (0.67, 1.81)	0.96 (0.73, 1.27)
lag 6	1.22 (0.94, 1.57)	1.35 (0.45, 4.05)	1.00 (0.86, 1.18)	1.07 (0.70, 1.65)	1.02 (0.78, 1.34)
lag 7	0.90 (0.70, 1.15)	2.29 (0.68, 7.76)	1.07 (0.91, 1.26)	0.77 (0.52, 1.12)	1.17 (0.91, 1.52)
	*Rockford, IL*	*Toledo, OH*			
lag 1	1.10 (0.67, 1.82)	0.97 (0.68, 1.39)			
lag 2	0.77 (0.42, 1.42)	0.70 (0.48, 1.03)			
lag 3	0.81 (0.44, 1.47)	1.11 (0.76, 1.63)			
lag 4	1.02 (0.61, 1.72)	0.64 (0.42, 0.96)			
lag 5	1.35 (0.84, 2.16)	1.02 (0.71, 1.46)			
lag 6	0.75 (0.40, 1.40)	1.01 (0.71, 1.45)			
lag 7	1.29 (0.79, 2.09)	1.64 (1.19, 2.25)			

## References

[1] Dufour A.P., Wymer L.J. (2006). Microbes, monitoring, and human health. Oceanography.

[2] Marion J.W., Lemeshow S., Buckley T.J. (2010). Association of gastrointestinal illness and recreational water exposure at an inland U.S. beach. Water Res..

[3] Wade T.J., Calderon R.L., Brenner K.P., Sams E., Beach M., Haugland R., Wymer L., Dufour A.P. (2008). High sensitivity of children to swimming-associated gastrointestinal illness: Results using rapid assay of recreational water quality. Epidemiology.

[4] Fleisher J.M., Kay D., Salmon R.L., Jones F., Wyer M., Godfree A.F. (1996). Marine waters contaminated with domestic sewage: Nonenteric illnesses associated with bather exposure in the United Kingdom. Am. J. Public Health.

[5] Wade T.J., Pai N., Eisenberg J.N.S., Colford J.M. (2003). Do US EPA water quality guidelines prevent gastrointestinal symptoms? A systematic review and meta analysis. Environ. Health Persp..

[6] U.S. Environmental Protection Agency (USEPA) (2003). Bacterial Water Quality Standards for Recreational Waters (Freshwater and Marine Waters).

[7] Efstratiou M.A. (2001). Managing coastal bathing water quality: The contribution of microbiology and epidemiology. Mar. Pollut. Bull..

[8] Marsalek J., Rochfort Q. (2003). Urban wet weather flows: Sources of fecal contamination impacting on recreational waters and threatening drinking water resources. J. Toxicol. Environ. Health.

[9] Ackerman D., Weisberg S.B. (2003). Relationship between rainfall and beach bacterial concentrations on Santa Monica Bay Beaches. J. Water Health.

[10] Olyphant G.A., Thomas J., Whitman R.L., Harper D. (2003). Characterization and statistical modeling of bacterial (*Escherichia coli*) outflows from watersheds that discharge into southern Lake Michigan. Environ. Monit. Assess..

[11] Byappanahallli M.N., Whitman R.L., Shively D.A., Nevers M.B. (2010). Linking non-culturable (qPCR) and culturable *Enterococci* densities with hydrometeorological conditions. Sci. Total Environ..

[12] Nevers M.B., Whitman R.L. (2011). Efficacy of monitoring and empirical predictive modeling at improving public health protection at Chicago beaches. Water Res..

[13] Whitman R.L., Nevers M.B. (2008). Summer *E. coli* patters and responses along 23 Chicago beaches. Environ. Sci. Technol..

[14] Wade T.J., Sams E., Brenner K.P., Haugland R., Chern E., Beach M., Wymer L., Rankin C.C., Love D., Li Q. (2010). Rapidly measured indicators of recreational water quality and swimming-associated illness at marine beaches: A prospective cohort study. Environ. Health.

[15] Pruss A. (1998). Review of epidemiological studies on health effects from exposure to recreational water. Int. J. Epidemiol..

[16] Curriero F.C., Patz J.A., Rose J.B., Lele S. (2001). The association between extreme precipitation and waterborne disease outbreaks in the United States, 1948–1994. Am. J. Public Health.

[17] Regional Climate Trends and Scenarios for the U.S. National Climate Assessment. Part 3. Climate of the Midwest U.S.. http://www.nesdis.noaa.gov/technical_reports/NOAA_NESDIS_Tech_Report_142-3-Climate_of_the_Midwest_U.S.pdf.

[18] Schuster C.J., Ellis A., Robertson W.J., Charron D.F., Aramini J.J., Marshall B.J. (2005). Medeiros. D.T. Infectious disease outbreaks related to drinking water in Canada, 1974–2001. Can. J. Public Health.

[19] Rose J.B., Daeschner S., Easterling D.R., Curriero F.C., Lele S., Patz J.A. (2000). Climate and waterborne disease outbreaks. Am. Water Works Assoc. J..

[20] Aramini J.J., Mclean M., Wilson J., Holt J., Copes R., Allen B., Sears W. (2000). Drinking water quality and health care utilization for gastrointestinal illness in greater Vancouver. Can. Commun. Dis. Rep..

[21] Egorov A.I., Naumova E.N., Tereschenko A.A., Kislitsin V.A., Ford T.E. (2003). Daily variations in effluent water turbidity and diarrhoeal illness in a Russian city. Int. J. Environ. Health Res..

[22] Schwartz J., Levin R., Goldstein R. (2000). Drinking water turbidity and gastrointestinal illness in the elderly of Philadelphia. J. Epidemiol. Commun. Health.

[23] Haley B.J., Cole D.J., Lipp E.K. (2009). Distribution, diversity, and seasonality of waterborne salmonella in a rural watershed. Appl. Environ. Microbiol..

[24] Jagai J.S., Castronovo D.A., Monchak J., Naumova E.N. (2009). Seasonality of cryptosporidiosis: A meta-analysis approach. Environ. Res..

[25] Eisenberg J.N.S., Seto E.Y.W., Colford J.M., Olivieri A., Spear R.C. (1998). An analysis of the Milwaukee cryptosporidiosis outbreak based on a dynamic model of the infection process. Epidemiology.

[26] Naumova E.N., Egorov A.I., Morris R.D., Griffiths J.K. (2003). The elderly and waterborne Cryptosporidium infection: Gastroenteritis hospitalizations before and during the 1996 Milwaukee Outbreak. Emerg. Infect. Dis..

[27] Wong M., Kumar L., Jenkins T.M., Xagoraraki I., Phanikuman M.S., Rose J.B. (2009). Evaluation of public health risks at recreational beaches in Lake Michigan via detection of enteric viruses and a human-specific bacteriological marker. Water Res..

[28] Dorfman M. (2006). Testing the Waters: A Guide to Water Quality at Vacation Beaches.

[29] Dorfman M., Rosselot K.S. (2010). Testing the Waters: A Guide to Water Quality at Vacation Beaches.

[30] Centers for Medicare and Medicaid Services. www.cms.gov.

[31] Morris R.D., Naumova E.N., Levin R., Munasinghe R.L. (1996). Temporal variation in drinking water turbidity and diagnosed gastroenteritis in Milwaukee. Am. J. Public Health.

[32] United States National Weather Service (NWS) (2010). Cooperative Observer Program.

[33] Kalkstein L.S., Valimont K.M. (1986). An evaluation of summer discomfort in the United States using a relative climatological index. Bull. Am. Meteorol. Soc..

[34] Steadman R.G. (1979). The assessment of sultriness. Part II: Effects of wind, extra radiation and barometric pressure on apparent temperature. J. App. Meteorol..

[35] Thirsty for Answers: Preparing for the Water-related impacts of Climate Change in American Cities. http://www.nrdc.org/water/files/thirstyforanswers.pdf.

[36] Haack S.K., Fogarty L.R., Wright C. (2003). *Escherichia coli* and *Enterococci* at beaches in the Grand Traverse Bay, Lake Michigan: Sources, characteristics, and environmental pathways. Environ. Sci. Technol..

[37] Scopel C.O., Harris J., McLellan S.L. (2006). Influence of nearshore water dynamics and pollution sources on beach monitoring outcomes at two adjacent Lake Michigan beaches. J. Great Lakes Res..

[38] McCullagh P., Nelder J.A. (1989). Generalized Linear Models.

[39] Dean C.B. (1992). Testing for overdispersion in Poisson and Binomial regression models. J. Am. Stat. Assoc..

[40] Schwartz J., Levin R., Goldstein R. (1997). Drinking water turbidity and pediatric use for gastrointestinal illness in Philadelphia. Epidemiology.

[41] Checkley W., Epstein L.D., Gilman R.H., Figueroa D., Cama R.I., Patz J.A. (2000). Effects of El Nino and ambient temperature on hospital admissions for diarrhoeal diseases in Peruvian children. Lancet.

[42] Fleury M., Charron D.F., Holt J.D., Allen O.B., Maarouf A.R. (2006). A time series analysis of the relationship of ambient temperature and common bacterial enteric infections in two Canadian provinces. Int. J. Biometeorol..

[43] Singh R.B.K., Hales S., de Wet N., Raj R., Hearnden M., Weinstein P. (2001). The influence of climate variation and change on diarrheal disease in the Pacific Islands. Environ. Health Perspect..

[44] Trinh C., Prabhakar K. (2007). Diarrheal diseases in the elderly. Clin. Geriatr. Med..

[45] Hastie T., Tibshirani R. (1986). Generalized additive models (with discussion). Stat. Sci..

[46] Hastie T., Tibshirani R. (1990). Exploring the nature of covariate effects in the proportional hazards model. Biometrics.

[47] Berkey C.S., Hoaglin D.C., Mosteller F., Colditz G.A. (1995). A random-effects regression model for meta-analysis. Stat. Med..

[48] Normand S.L.T. (1999). Tutorial in biostatistics. Meta-analysis: Formulating, evaluating, combining, and reporting. Stat. Med..

[49] United States Census Bureau American Fact Finder. http://factfinder2.census.gov/faces/nav/jsf/pages/index.xhtml.

[50] Drayna P., McLellan S.L., Simpson P., Li S., Gorelick M.H. (2010). Association between rainfall and pediatric emergency department visits for acute gastrointestinal illness. Environ. Health Persp..

[51] Sampson R.W., Swiatnicki S.A., McDermott C.M., Kleinheinz G.T. (2006). The effects of rainfall on *Escherichia coli* and total coliform levels at 15 lake superior recreational beaches. Water Res. Manage. J..

[52] Charron D.F., Thomas M.K., Waltner-Toews D., Aramini J.J., Edge T., Kent R.A., Maarouf A., Wilson J. (2004). Vulnerability of waterborne diseases to climate change in Canada: A review. J. Toxicol. Environ. Health.

[53] Ford T.E. (1999). Microbiological safety of drinking water: United States and global perspectives. Environ. Health Persp..

[54] Nowcasting and Forecasting of Beach Bacteria Concentration Using EPA’s Virtual Beach Software. http://adsabs.harvard.edu/abs/2007AGUSMOS23G..05F.

[55] Nevers M.B., Whitman R.L. (2005). Nowcast modeling of *Escherichia coli* concentrations at multiple urban beaches of southern Lake Michigan. Water Res..

[56] Intergovernmental Panel on Climate Change (IPCC) (2012). Managing the Risks of Extreme Events and Disasters to Advance Climate Change Adaptation Summary for Policymakers.

[57] Sterk A., Schijven J., de Nijs T., de Roda Husman A.M. (2013). Direct and indirect effects of climate change on the risk of infection by water-transmitted pathogens. Environ. Sci. Technol..

